# The Folly of Packing in Post-Partum Hemorrhage

**Published:** 1911-10

**Authors:** Wm. Gillespie


### The Folly of Packing in Post-Partum Hemorrhage

For years packing the uterus has been advocated as an effective treatment of post-partum hemorrhage. Beautiful pictures have been introduced into our modern text-books to illustrate the methods to be pursued; and the medical student goes out into practice feeling that there is one thing at least which he thoroughly understands.

It is the object of this discussion to point out some of the weaknesses, the absurdities and the uselessness of this method of treatment.

In the real typical post-partum flooding, nothing could be more absurd than to attempt this method of controlling the hemorrhage. With the flood-gates open and the stream of blood gushing to the full capacity of the vagina and squirting to the knees of the patient, before the necessary preliminary steps could be taken to begin to pack, the life of the patient would be extinct. There is but one method of treatment which the writer has found to be prompt enough to be safe and effective, namely, carrying one hand into the uterus and making vigorous friction with the other upon its fundus. Time can not even be spared for preliminary care as to asepsis. An antiseptic solution should always be within easy reach, and if the internal hand is dipped into this the chances of infection are lessened. Ergot should be pumped under the skin in full doses until the uterus is ergotized, and a stream of hot water may with advantage be carried up alongside the internal hand, but these things must be prepared by others. The one who temporarily stems the tide must not for an instant remove either hand from its appointed task. The internal hand should, before its removal, look for any remnants of placenta, placenta succenturiata or submucous fibroid. The removal of placental remnants will take away the cause of hemorrhage, the discovery of a submucous fibroid will give warning of the danger of subsequent hemorrhage or sloughing. When uterine contraction does not stop the flow, pull down the uterus and look for and suture the laceration in its tissue, which is almost certainly present.

There are many uncertainties in obstetric practice, but of this one may be quite sure: He who believes he has saved a life, in real post-partum flooding, by tamponing the uterus deludes himself, for the fate of such cases is often decided in the first minute of the flooding.

The continuous hemorrhage of moderate amount is almost certainly due to some of the conditions we have enumerated, and will yield to the treatment suggested.

Our forefathers knew that the only safe uterus was an empty one. One modern writer suggests a plan of treatment which ignores this knowledge, which distends the organ so that it can not retract when retraction is our only real safety, and which will stimulate contraction, to be followed by relaxation and more hemorrhage. If the uterus was a hollow, inelastic cavity, one might reasonably expect by tight packing to control hemorrhage, but the organ has, until a few minutes ago, been distended to a size sufficient to accommodate a hemorrhage of sufficient magnitude to destroy life, and the larger the uterine cavity the more copiously will the blood flow.

At a little later period a hemorrhage from a disintegrating fibroid may well be controlled by gauze packing until preparations can be made for its removal.

If the truth were known, we think it quite certain that the reputation of this method has been earned by its apparent success in cases of overlooked deep cervical lacerations, and from its being used in uteri where, from the character of the labor, the attendant feared that hemorrhage would occur. As hemorrhage occurs in only a small minority of the cases which present its predisposing factors, it is easy to build up a false reputation for any treatment employed to combat its occurrence. In the early months of pregnancy it would not be so difficult to control hemorrhage by this method, for the uterus is small, and may be filled by gauze to its full capacity, yet in the experience of the writer it has never seemed to be indicated. Many years ago he imbibed the doctrine that in abortion one thing was certain, namely, the empty uterus does not bleed. Therefore, as long as bleeding continues, he feels safe in assuming that some of the products of conception remain in the uterus, and with their removal hemorrhage has always ceased. That this is not his individual experience is amply attested by the testimony of others who assume as true the doctrine quoted above.

Uterine packing serves a useful purpose in the presence of hemorrhage before labor or abortion has occurred, but we wish to serve the interests of humanity by assuring the inexperienced practitioner of the folly of such methods in post-partum hemorrhage at any period of gestation.—Wm. Gillespie, M. D., in *The Lancet-Clinic.*

